# Changes in Sensory Phenomena, Tics, Obsessive–Compulsive Symptoms, and Global Functioning of Tourette Syndrome: A Follow-Up After Four Years

**DOI:** 10.3389/fpsyt.2020.00619

**Published:** 2020-06-30

**Authors:** Yukiko Kano, Miyuki Fujio, Namiko Kaji, Natsumi Matsuda, Maiko Nonaka, Toshiaki Kono

**Affiliations:** ^1^ Department of Child Neuropsychiatry, Graduate School of Medicine, The University of Tokyo, Tokyo, Japan; ^2^ Department of Child Psychiatry, The University of Tokyo Hospital, Tokyo, Japan; ^3^ Course of Clinical Psychology, Graduate School of Education, The University of Tokyo, Tokyo, Japan; ^4^ Department of Psychology, Faculty of Liberal Arts, Teikyo University, Tokyo, Japan; ^5^ Office for Mental Health Support, Division for Counseling and Support, The University of Tokyo, Tokyo, Japan; ^6^ Department of Developmental Psychology, Faculty of Human Studies, Shirayuri University, Tokyo, Japan; ^7^ Department of Community Mental Health and Law, National Institute of Mental Health, National Center of Neurology and Psychiatry, Tokyo, Japan

**Keywords:** sensory phenomena, tics, Tourette syndrome, obsessive–compulsive symptoms, global functioning

## Abstract

Sensory phenomena and related features of Tourette syndrome are related to poorer quality of life. Therefore, sensory phenomena might also have a negative impact on global functioning. However, the influence of sensory phenomena, tics, and obsessive–compulsive symptoms (OCS) on global functioning after several years of usual treatment has not been investigated. Twenty out of 45 Japanese patients with Tourette syndrome who had previously undergone an evaluation of these clinical features were assessed again after an average of four years. We conducted a panel of assessments for premonitory urges, broader sensory phenomena, tic severity, OCS, and global functioning. Based on Pearson’s correlation coefficient, current global functioning was significantly negatively correlated with previous tics and marginally negatively correlated with previous broader sensory phenomena. Current global functioning was marginally correlated with change in tics. Change in global functioning was significantly correlated with change in OCS and marginally correlated with change in premonitory urges. Due to the small sample size, it was not possible to use a multiple regression analysis to conclude that sensory phenomena, tics, and OCS predict global functioning in adolescents and adults with TS. However, it was suggested that further investigation of this relationship would be meaningful.

## Introduction

Tourette syndrome (TS) is a childhood-onset neurodevelopmental disorder characterized by multiple motor and vocal tics (1). Aside from tics, the crucial symptoms of TS are “sensory phenomena,” which include urges to move that often precede tics (i.e., premonitory urges) and a strong desire to have things be “just right” (2).

Sensory phenomena in TS have often been investigated in relation to frequent comorbid symptoms such as obsessive–compulsive symptoms (OCS) and global functioning. For instance, one study indicated that sensory phenomena, especially premonitory urges, were significantly correlated with vocal tics and OCS in adults with TS (3). Another study showed that sensory phenomena were significantly correlated with clinical symptoms, particularly OCS and anxiety (4). Among sensory phenomena, “just right” perception is an important feature of tic-related OCD. Our previous studies showed that premonitory urges and broader sensory phenomena including “just right” perception are different in terms of their relationships with OCS dimensions (5) and clinical course after Deep Brain Stimulation (6).

Sensory phenomena, especially premonitory urges were significantly and negatively correlated with quality of life (QOL) in previous research (3, 4). QOL is evaluated as one’s perception of broader health, whereas global functioning represents psychological, social, and occupational functions assessed by behaviors. Despite this difference between QOL and global functioning, the relationship between sensory phenomena and global functioning seems to be similar to that between sensory phenomena and QOL. In a previous study, we found that both premonitory urges and broader sensory phenomena were significantly positively correlated with total tics, vocal tics, and OCS, while premonitory urges were significantly and negatively correlated with global functioning (5).

One study showed that premonitory urges, tic severity, and family history of TS in childhood were predictors of poorer QOL in adults with TS (7). However, there have been no investigations of the influence of whole sensory phenomena and their related features (e.g., tics and OCS) on global functioning during the clinical course of TS. We believe that such an investigation would provide a deeper understanding of TS as well as better treatment and support. Thus, in this study, we described changes in sensory phenomena as well as tics, OCS, and global functioning after several years of usual treatment for TS. Based on these data, we examined whether previous sensory phenomena, tics, and OCS would predict current global functioning. We also examined whether changes in sensory phenomena, tics, and OCS over the course of treatment would predict current global functioning as well as change in global functioning. Based on previous findings, we hypothesized that more severe previous sensory phenomena, tics, and OCS would linearly predict poorer current global functioning.

## Methods

### Participants

Participants were recruited from the 45 patients with TS who had participated in our previous study (5). Out of the 45 patients, we asked 22 to participate in the current study, but two patients did not accept our request. Of the remaining 23 patients, we were unable to ask 15 to participate because they had changed hospitals or had completed or dropped out of treatment. An additional eight patients were not asked to participate because they visited the hospital rarely or irregularly (n = 5), had undergone deep brain stimulation (n = 2), or were in an unstable condition (n = 1). Therefore, the participants of the study consisted of 20 patients with TS (16 men and 4 women; age range, 17–53 years; mean = 30.2; SD = 11.2) who had fully completed all study instruments. All participants had been diagnosed with Tourette’s disorder according to the Diagnostic and Statistical Manual of Mental Disorders, Fourth Edition, Text Revision (DSM-IV-TR) (8). Participants also met the DSM-5 (fifth edition) criteria for Tourette’s disorder (1). The participants were recruited between January 2014 and May 2015, and the mean time since previous study participation was 4.0 years (SD = 0.61, range: 3–5 years). Data obtained from these participants in the previous study were also used in analyses.

The Institutional Ethical Committee of the University of Tokyo Hospital approved this study. Written informed consent was obtained from all adult participants and the parents of participants aged 19 years old or younger. Psychologists with clinical experience of TS and sufficient ability to perform an assessment following instrument administration training conducted all the interviews.

### Instruments

To facilitate comparison between the studies, the same instruments used in our previous study to assess sensory phenomena, tics, and global functioning (5) were utilized in the current study. Assessment of sensory phenomena included administration of the University of São Paulo Sensory Phenomena Scale (USP-SPS) (5, 9) and the Premonitory Urge for Tics Scale (PUTS) (10). The USP-SPS is a clinician-rated scale that assesses presence or absence of sensory phenomena in five types, including “just right” perception, and measures severity of broader sensory phenomena on three ordinal scales focusing on frequency, distress, and interference, with six anchor points. The USP-SPS total score (0–15) is obtained by combining these scores. The PUTS is a 9-item self-report scale that measures severity of premonitory urges for tics. The PUTS total score (9–36) is obtained by summing the scores for all items.

Tics were evaluated using the Yale Global Tic Severity Scale (YGTSS) (11, 12), which is a clinician-rated scale that measures severity of motor and vocal tics as well as impairment due to tics. The YGTSS total tics score (0–50) is obtained by summing the scores for motor and vocal tics on five ordinal scales focusing on number, frequency, intensity, complexity, and interference, with six anchor points. The YGTSS global severity score (0–100) is obtained by summing the total tics score (0–50) and the impairment score (0–50). Global functioning was evaluated using the Global Assessment of Functioning (GAF) scale (8).

Comorbid obsessive–compulsive disorder (OCD) and attention-deficit/hyperactivity disorder (ADHD) were diagnosed according to the DSM-IV-TR criteria, in addition to TS. Information about medication use was obtained from patients’ psychiatrists and medical records. In the current study, OCS were evaluated using the Yale-Brown Obsessive–Compulsive Scale (Y-BOCS) (13, 14), which is a clinician-rated scale that measures severity of obsessions and compulsions on five ordinal scales with five anchor points. The Y-BOCS total score (0–40) was obtained by summing the scores for the obsessions (0–20) and compulsions (0–20) scales.

### Statistical Analyses

All statistical analyses were performed using PASW Statistics 18.0 (i.e., SPSS). The Shapiro-Wilk test showed that the assessment scores in this study were normally distributed, except for the current YGTSS impairment score. A paired t-test was used to compare the scores on all instruments between the previous and current studies. In addition, the Wilcoxon rank sum test was used for comparison of YGTSS impairment score between the two studies. After examining the associations between previous and current sensory phenomena, tics, and OCS, the correlations of current global functioning with previous sensory phenomena, tics, and OCS were examined. In all the association analyses, total score was used for USP-SPS, global severity score for YGTSS, and total score for Y-BOCS. The score changes in sensory phenomena, tics, OCS, and global functioning were defined as the differences between current and previous scores. The interrelationships between the score changes in clinical characteristics and current global functioning, as well as the score change in global functioning, were also examined. For these examinations, Pearson’s correlation coefficients were calculated. The standard *p*<0.05 level of significance was used. When we examined correlations between previous and current USP-SPS, PUTS, YGTSS, and Y-BOCS in *Correlations Between Previous and Current Sensory Phenomena, Tics, and OCS*; correlations between previous USP-SPS, PUTS, YGTSS, and Y-BOCS, and current GAF in *Correlations Between Previous Sensory Phenomena, Tics, and OCS, and Current Global Functioning*; correlations between change in USP-SPS, PUTS, YGTSS, and Y-BOCS, and current GAF in *Correlations Between Changes in Sensory Phenomena, Tics, and OCS, and Current Global Functioning*; and correlations between changes in USP-SPS, PUTS, YGTSS, and Y-BOCS, and change in GAF in *Correlations Between Changes in Sensory Phenomena, Tics, and OCS, and Change in Global Functioning*; the Bonferroni correction was applied and the level of significance changed to p<0.0125.

## Results

Before analyzing data of the current study, we compared age, sensory phenomena, tics, OCS, and global functioning in the previous study between the 20 current study participants and the 25 non-participants by an independent sample t-test. Fisher’s exact test was used to compare gender differences between the two groups. Although we found that Y-BOCS total scores were significantly higher in the latter group (*p* = 0.02), no other significant differences were found (*p* = 0.12–0.93).

### Description of Sensory Phenomena, Tics, OCS, Global Functioning, Comorbidity, and Medication in the Current Study

In the current study, the mean total scores for the USP-SPS and PUTS were 5.0 and 10.2, respectively (**Table 1**). Out of five types of sensory phenomena, rate of Energy build-up decreased the most between two studies (**Table 2**). Counting change in each person, the proportion without change was highest at 95.2% for Auditory “just-right” perception, and lowest at 52.4% for Tactile sensation.

**Table 1:** Participants’ demographic and clinical characteristics.

		Previous	Current	*t*-value	*p*-value
Age (years)		26.1 (11.2; 13–49)	30.2 (11.2; 17–53)	21.5	0.00
USP-SPS	Total	6.2 (3.1; 0–12)	5.0 (3.2; 0–12)	−1.7	0.11
	Frequency	3.0 (1.6; 0–5)	2.3 (1.5; 0–5)	−1.4	0.18
	Distress	1.9 (1.0; 0–4)	1.7 (1.1; 0–4)	−1.2	0.23
	Interference	1.3 (1.3; 0–4)	1.0 (1.2; 0–4)	−1.2	0.23
PUTS		10.6 (6.6; 1–25)	10.2 (6.1; 0–26)	−0.3	0.74
YGTSS	Total tics	21.3 (8.1; 7–37)	20.9 (9.0; 8–37)	−0.4	0.76
	Motor tics	10.1 (6.2; 0–22)	9.9 (7.4; 0–22)	−0.3	0.75
	Vocal tics	11.2 (4.2; 4–19)	11.1 (4.6; 0–17)	−0.1	0.89
	Impairment	22.0 (11.1; 0–40)	21.0 (13.3; 0–40)	−0.4	0.67 ^a^
	Global severity	43.3 (17.9; 7–77)	41.9 (21.1; 8–77)	−0.4	0.66
Y-BOCS	Total	11.7 (8.9; 0–29)	7.8 (7.3; 0–25)	−2.8	0.01
	Obsessions	5.7 (4.4; 0–14)	4.2 (4.0; 0–14)	−1.4	0.17
	Compulsions	6.1 (5.4; 0–15)	3.6 (3.4; 0–11)	−3.0	0.008
GAF		64.7 (11.1; 45–86)	62.4 (10.5; 43–83)	−1.1	0.28

Data reported as: Mean (SD; range).

USP-SPS, University of São Paulo Sensory Phenomena Scale; PUTS, Premonitory Urge for Tics Scale; YGTSS, Yale Global Tic Severity Scale; Y-BOCS, Yale-Brown Obsessive–Compulsive Scale; GAF, Global Assessment of Functioning.

^a^When the Wilcoxon rank sum test was used for comparison between the two studies, the p value became 0.642.

**Table 2:** Participants’ number with each type of sensory phenomena.

	Previous	Current
A. Physical sensation	14 (70%)	12 (60%)
A1. Tactile	10 (50%)	8 (40%)
A2 Muscle-joint	8 (40%)	9 (45%)
B. “Just-right” perception	11 (55%)	10 (50%)
B1. Visual	5 (25%)	5 (25%)
B2. Auditory	5 (25%)	4 (20%)
B3. Tactile	7 (35%)	7 (35%)
C. Feeling of incompleteness	8 (40%)	6 (30%)
D. Energy build-up	9 (45%)	4 (20%)
E. Urge only	11 (55%)	9 (45%)

The mean scores on the YGTSS were 20.9 for total tics, 21.0 for impairment due to tics, and 41.9 for global severity. The mean GAF score was 62.4. None of these scores differed significantly between the current and previous studies. The mean total score of the Y-BOCS was 7.8 in the current study, which was significantly lower than that in the previous study (*p* = 0.01).

In the current study, six (30%) participants had comorbid OCD and four (20%) had comorbid ADHD. All participants were taking some form of psychotropic drug. Specifically, all participants were on antipsychotics, including aripiprazole (*n* = 12), risperidone (*n* = 4), and haloperidol (n = 4). Nineteen were on other drugs, including antidepressants (*n* = 13, of which 11 were on selective serotonin reuptake inhibitors), clonazepam (*n* = 6), anxiolytics (*n* = 2), and clonidine (*n* = 1). There were only slight differences in medication between the current and previous studies, although the number of participants on antidepressants increased from 9 to 13.

### Correlations Between Previous and Current Sensory Phenomena, Tics, and OCS

Previous USP-SPS total scores and PUTS total scores were significantly correlated with previous YGTSS global severity scores and Y-BOCS total scores (*p*<0.001, *p* = 0.026, respectively, for USP-SPS; *p<*0.001, *p* = 0.002, respectively, for PUTS). Current USP-SPS total scores were significantly correlated with current YGTSS global severity scores (*p* = 0.009), while current PUTS total scores were marginally correlated with current YGTSS global severity scores (*p* = 0.024). Both current USP-SPS total scores and PUTS total scores were significantly correlated with current Y-BOCS total scores (*p* = 0.002, *p* = 0.002, respectively). Previous USP-SPS total scores were significantly correlated with current Y-BOCS total scores (*p* = 0.006) and marginally correlated with current YGTSS global severity scores (*p* = 0.048). Previous PUTS total scores were marginally correlated with current Y-BOCS total scores (*p* = 0.019).

### Correlations Between Previous Sensory Phenomena, Tics, and OCS, and Current Global Functioning

Current GAF scores were significantly negatively correlated with previous YGTSS global severity scores (*p* = 0.005) and marginally negatively correlated with USP-SPS total scores (*p* = 0.023) (**Table 3**).

**Table 3:** Correlations between previous sensory phenomena, tics, and OCS, and current global functioning.

	Current GAF	
	*r*	*p*
Previous USP-SPS	−0.505	0.023
Previous PUTS	−0.327	0.186
Previous YGTSS	−0.602	0.005
Previous Y-BOCS	−0.373	0.127

USP-SPS, University of São Paulo Sensory Phenomena Scale; PUTS, Premonitory Urge for Tics Scale; YGTSS, Yale Global Tic Severity Scale; Y-BOCS, Yale-Brown Obsessive–Compulsive Scale; GAF, Global Assessment of Functioning; OCS, Obsessive–Compulsive symptoms.

### Correlations Between Changes in Sensory Phenomena, Tics, and OCS, and Current Global Functioning

Current GAF scores were marginally correlated with the change in YGTSS global severity scores (*p* = 0.042; **Table 4**).

**Table 4:** Correlations between changes in sensory phenomena, tics, and OCS, and current global functioning.

	Current GAF	
	*r*	*p*
Change in USP-SPS	0.073	0.759
Change in PUTS	0.289	0.229
Change in YGTSS	0.458	0.042
Change in Y-BOCS	0.192	0.459

USP-SPS, University of São Paulo Sensory Phenomena Scale; PUTS, Premonitory Urge for Tics Scale; YGTSS, Yale Global Tic Severity Scale; Y-BOCS, Yale-Brown Obsessive–Compulsive Scale; GAF, Global Assessment of Functioning; OCS, Obsessive–Compulsive symptoms.

### Correlations Between Changes in Sensory Phenomena, Tics, and OCS, and Change in Global Functioning

The change in GAF scores was significantly correlated with the changes in Y-BOCS total scores (*p* = 0.009) and marginally correlated with the change in PUTS total scores (*p* = 0.023) (**Table 5**).

**Table 5:** Correlations between changes in sensory phenomena, tics, and OCS, and change in global functioning.

	Change in GAF	
	*r*	*p*
Change in USP-SPS	−0.264	0.275
Change in PUTS	0.518	0.023
Change in YGTSS	0.383	0.105
Change in Y-BOCS	0.630	0.009

USP-SPS, University of São Paulo Sensory Phenomena Scale; PUTS, Premonitory Urge for Tics Scale; YGTSS, Yale Global Tic Severity Scale; Y-BOCS, Yale-Brown Obsessive–Compulsive Scale; GAF, Global Assessment of Functioning; OCS, Obsessive–Compulsive symptoms.

## Discussion

The adolescents and adults with TS in this study demonstrated significant improvement in OCS after an average of four years of usual treatment, although their sensory phenomena, tics, and global functioning showed little change. In these participants, sensory phenomena were significantly correlated with tics and OCS in the past. Broader sensory phenomena were significantly correlated with tics and OCS, and premonitory urges were significantly correlated with OCS in the present. These findings were similar to our previous study (5). Broader sensory phenomena in the past were significantly correlated with OCS in the present. The influence of previous sensory phenomena on current OCS seems to be slightly stronger than that on current tics.

We found that previous tics were significantly negatively correlated with current global functioning and previous broader sensory phenomena were marginally correlated with current global functioning, based on Pearson’s correlation coefficient. These results were mostly consistent with our expectations, particularly the relationship between previous tics and current global functioning. On the other hand, previous premonitory urges were not correlated with current global functioning. This seems to be different from a previous study that found that premonitory urges predicted QOL (7), although a similar relationship between premonitory urges and global functioning was expected. A possible reason for this difference is related to study participants. Specifically, in Cavanna et al.’s study, the participants were children or adolescents at the first assessment, and they had poor or insufficient recognition of premonitory urges, whereas all the participants in our study had obtained sufficient recognition of their premonitory urges.

We did find that change in tics was marginally related to current global functioning. Improvement of tics would alleviate tic-related impairment of life, and then improve global functioning. In a previous study, subjective satisfaction with tic control was positively correlated with life satisfaction and QOL (15). If tic control results in improvement of tics, increased life satisfaction might have a positive impact on global functioning. We found that change in premonitory urges was marginally related to change in global functioning, although the relationship between previous premonitory urges and current global functioning was not significant. The difference between the two findings suggests that when change in premonitory urges is large, it may influence global functioning. In addition, significant improvement of OCS over the clinical course might affect the relationship between changes in OCS and global functioning.

The current study has several limitations. First, all the participants had regularly visited a single specialty clinic for TS and related disorders for several years, which led to a small, potentially biased sample. Moreover, due to the small sample size, sufficient statistical power was not achieved in certain instances. In addition, we applied Bonferroni correction only for association analyses. Second, we could not examine influence by comorbid ADHD, depression, anxiety, and other symptoms, although previous studies have suggested it (16) and recommended comprehensive assessment (17). Third, the possible influence of change in medication during the clinical course on clinical characteristics and global functioning was not examined. Finally, the small sample size did not allow use of a multiple regression analysis, which would be necessary to draw firm conclusions.

Due to these limitations, it was not possible to conclude that sensory phenomena, tics, and OCS predict global functioning in adolescents and adults with TS; however, the findings suggested that further investigation of this relationship would be meaningful. During the clinical course, premonitory urges and broader sensory phenomena might have slightly different effect on global functioning. In the future, researchers might recruit a larger number of patients to further clarify these findings.

## Data Availability Statement

The datasets generated for this study are available on request to the corresponding author.

## Ethics Statement

The Institutional Ethical Committee of the University of Tokyo Hospital approved this study. Written informed consent was obtained from all adult participants and the parents of participants aged 19 years old or younger.

## Author Contributions 

YK was in charge of research design and writing the manuscript. MF was in charge of data collection and analysis. NK was in charge of data collection. NM was in charge of data analysis and reviewing the manuscript. MN and TK were involved in discussion about the research design and reviewed the manuscript.

## Funding 

The present study was partly supported by a Grant for Comprehensive Research on Disability, Health and Welfare [H26-Seishin-Ippan007] from the Ministry of Health, Labour and Welfare in Japan, and an Intramural Research Grant (23-1) for Neurological and Psychiatric Disorders of the National Center of Neurology and Psychiatry (NCNP). Further support was received from a Grant-in-Aid for Scientific Research on Innovative Areas (Adolescent Mind & Self-Regulation) [grant number 26118704], a Grant-in-Aid for Scientific Research (C) [15K09859] from the Ministry of Education, Culture, Sports, Science and Technology in Japan, the Disabled Overall Welfare Promotion Business (Designated Subject #16 in the fiscal 2018, PI: Inagaki M) of the Ministry of Health, Labour and Welfare in Japan, and Health, Labour and Welfare Sciences Research Grants, Comprehensive Research on Disability 19GC1001.

## Conflict of Interest

The authors declare that the research was conducted in the absence of any commercial or financial relationships that could be construed as a potential conflict of interest.

## References

[1] American Psychiatric Association Diagnostic and Statistical Manual of Mental Disorders. 4th Edn Washington DC: Author (2000). text revision.

[2] Houghton DC Capriotti MR Conelea CA Woods DW Sensory phenomena in Tourette syndrome: Their role in symptom formation and treatment. Curr Dev Disord Rep (2014) 1:245–51. 10.1007/s40474-014-0026-2 PMC4381438 25844305

[3] Crossley E Cavanna AE Sensory phenomena: Clinical correlates and impact on quality of life in adult patients with Tourette syndrome. Psychiatry Res (2013) 209:705–10. 10.1016/j.psychres.2013.04.019 23684051

[4] Eddy CM Cavanna AE Premonitory urges in adults with complicated and uncomplicated Tourette syndrome. Behav Modif (2014) 38:264–75. 10.1177/0145445513504432 24042069

[5] Kano Y Matsuda N Nonaka M Fujio M Kuwabara H Kono T Sensory phenomena related to tics, obsessive-compulsive symptoms, and global functioning in Tourette syndrome. Compr Psychiatry (2015) 62:141–6. 10.1016/j.comppsych.2015.07.006 26343478

[6] Kano Y Matsuda N Nonaka M Fujio M Kono T Kaido T Sensory phenomena and obsessive-compulsive symptoms in Tourette syndrome following deep brain stimulation: Two case reports. J Clin Neurosci (2018) 56:199–201. 10.1016/j.jocn.2018.06.046 30042071

[7] Cavanna AE David K Orth M Robertson MM Predictors during childhood of future health-related quality of life in adults with Gilles de la Tourette syndrome. Eur J Paediatr Neurol (2012) 16:605–12. 10.1016/j.ejpn.2012.02.004 22381812

[8] American Psychiatric Association Diagnostic and Statistical Manual of Mental Disorders. 5th Edn Arlington, VA: American Psychiatric Association (2013).

[9] Rosario MC Prado HS Borcato S Diniz JB Shavitt RG Hounie AG Validation of the University of São Paulo Sensory Phenomena Scale: Initial psychometric properties. CNS Spectr (2009) 14:315–23. 10.1017/S1092852900020319 19668122

[10] Woods DW Piacentini J Himle MB Chang S Premonitory Urge for Tics Scale (PUTS): Initial psychometric results and examination of the premonitory urge phenomenon in youths with tic disorders. J Dev Behav Pediatr (2005) 26:397–403. 10.1097/00004703-200512000-00001 16344654

[11] Inoko K Nishizono-Maher A Tani S Kano Y Kishimoto J Hayakawa N Reliability and validity of a Japanese version of the Yale Global Tic Severity Scale: A preliminary study. Jpn J Child Adol Psychiatry (2006) 47:38–48.

[12] Leckman JF Riddle MA Hardin MT Ort S II Swartz KL Stevenson J The Yale Global Tic Severity Scale: Initial testing of a clinician-rated scale of tic severity. J Am Acad Child Adolesc Psychiatry (1989) 28:566–73. 10.1097/00004583-198907000-00015 2768151

[13] Goodman WK Price LH Rasmussen SA Mazure C Fleischmann RL Hill CL The Yale-Brown Obsessive Compulsive Scale. I. Development, use, and reliability. Arch Gen Psychiatry (1989) 46:1006–11. 10.1001/archpsyc.1989.01810110048007 2684084

[14] Nakajima T Nakamura M Taga C Yamagami S Kiriike N Nagata T Reliability and validity of the Japanese version of the Yale-Brown Obsessive-Compulsive Scale. Psychiatry Clin Neurosci (1995) 49:121–6. 10.1111/j.1440-1819.1995.tb01875.x 8726128

[15] Matsuda N Kono T Nonaka M Fujio M Kano Y Self-initiated coping with Tourette’s syndrome: Effect of tic suppression on QOL. Brain Dev (2016) 38:233–41. 10.1016/j.braindev.2015.08.006 26360257

[16] Evans J Seri S Cavanna AE The effects of Gilles de la Tourette syndrome and other chronic tic disorders on quality of life across the lifespan: A systematic review. Eur Child Adolesc Psychiatry (2016) 25:939–48. 10.1007/s00787-016-0823-8 PMC4990617 26880181

[17] O’Hare D Helmes E Reece J Eapen V McBain K The differential impact of Tourette’s syndrome and comorbid diagnosis on the quality of life and functioning of diagnosed children and adolescents. J Child Adolesc Psychiatr Nurs (2016) 29:30–6. 10.1111/jcap.12132 26991016

